# GPCR Conformations: Implications for Rational Drug Design

**DOI:** 10.3390/ph4010007

**Published:** 2010-12-23

**Authors:** Abby L. Parrill, Debra L. Bautista

**Affiliations:** 1 Department of Chemistry, The University of Memphis, Memphis, TN 38152, USA; 2 Christian Brothers High School, 5900 Walnut Grove Road, Memphis, TN 38120, USA; E-Mail: dbautista@cbhs.org (D.L.B.)

**Keywords:** G protein-coupled receptor, GPCR, structure, crystallography, NMR, modeling

## Abstract

G protein-coupled receptors (GPCRs) comprise a large class of transmembrane proteins that play critical roles in both normal physiology and pathophysiology. These critical roles offer targets for therapeutic intervention, as exemplified by the substantial fraction of current pharmaceutical agents that target members of this family. Tremendous contributions to our understanding of GPCR structure and dynamics have come from both indirect and direct structural characterization techniques. Key features of GPCR conformations derived from both types of characterization techniques are reviewed.

## 1. Introduction

G protein-coupled receptors (GPCRs) are a large family of integral membrane proteins involved in the transduction of cellular signaling. Analysis of the human genome suggested about 950 human GPCR sequences [1]. An alternate analysis utilizing 200 published GPCRs to seed searches of the human genome database identified 802 unique human GPCRs after duplicate removal [2]. These estimates are supported by 1,426 Swiss-Prot and TrEMBL entries representing full-length human GPCR sequences referenced in the GPCRdb [3]. The latter list includes redundancies due to multiple depositions of the same sequence, thus 800-950 is a more accurate estimate of the number of human GPCR sequences. These receptors play essential roles in the action of hormones, neurotransmitters, growth factors and the immune system. Considering the prevalence of GPCRs and their essential roles in diverse biological functions, it is not surprising that approximately 25-50% of drugs act on GPCRs (variability stems from whether percentages are calculated on sales or drug identity) [4,5].

The large number of GPCR sequences has stimulated several classification efforts. The earliest classification system still in common use identifies six superfamilies, or clans, labeled A-F based on GPCRs in multiple species [6]. A more recent classification developed based on phylogenetic analysis of human GPCR sequences produced five families named based on key family members. These five families are glutamate (G), rhodopsin (R), adhesion (A), frizzled/taste2 (F) and secretin (S), referred to in aggregate as GRAFS [2] Several differences distinguish these systems. First, the D and E superfamilies of the A-F system contain no human homologs [7] instead including two classes of yeast pheromone receptors [8] Second, the A-F system combines the secretin and adhesion receptors of the GRAFS system into superfamily B [7] Despite these differences, both classification systems share some key similarities. In particular, both systems have large families of sequences similar to rhodopsin, classified as superfamily A in the A-F system or R in the GRAFS system. This is the largest family in both classification systems, and is characterized by several well-conserved sequence motifs and an agonist binding site typically located within the transmembrane domain (TM). The A-F system will be used throughout this review.

GPCRs exhibit limited conservation of structural features as a superfamily, with a more extensive set of conserved features occurring within the classes identified either by sequence similarity or by phylogenetic analysis. The most conserved feature is a topology characterized by an extracellular N-terminus, seven membrane-spanning alpha helices, and an intracellular C-terminus. The class A (rhodopsin-like) GPCRs additionally share several conserved sequence motifs. The sequence motif (E/D)R(Y/H) occurs frequently at the intracellular end of TM3 in these receptors. The role of this motif in receptor conformation and activation has been extensively reviewed, and two basic phenotypes were observed to occur as a consequence of mutations to this motif [9] The first phenotype is characterized by receptors that become constitutively active upon non-conservative mutation of the acidic residue, but show little change in agonist binding or G protein coupling upon mutation of the arginine. The second phenotype shows no constitutive activation due to mutation of the acidic residue, but shows disrupted agonist binding upon mutation of the arginine. Thus this motif clearly plays an important, but incompletely conserved, functional role in the class A GPCRs. A second highly conserved motif in the class A GPCRs is the NPxxY motif near the intracellular end of TM7. This sequence motif was identified as providing flexibility that could serve as a hinge during conformational changes [10] The crystal structures of rhodopsin [11], the β2-aderenoceptor [12-14], the β1-aderenoceptor [15], and the adenosine A2a receptor [16] all demonstrate water-mediated interactions between this motif and the conserved aspartate residue in TM2. Mutational analysis in the M1 muscarinic receptor [17] and spectroscopic studies of fluorescein-bound rhodopsin [18] indicate that this interaction plays a role in signal transduction. While class A has been subject to more intense study, class C GPCR members share a long amino terminal sequence preceding the first transmembrane alpha helical domain. This amino terminal domain is responsible for binding ligand, receptor dimerization, and plays an integral role in signal transduction [19]. Numerous crystallographic structures have been reported for isolated and dimeric amino-terminal domains of class C GPCRs [20-22] (the venus-flytrap domains). Class B GPCR members also share a large extracellular domain at the amino terminus, which drives ligand recognition and subsequent interactions with the transmembrane domain during activation. Many examples of ligand-bound extracellular domains of class B GPCRs have also been crystallized [23-30] Crystallographic structures of complete GPCRs, including the transmembrane portion, have been much more limited.

Determination of membrane protein structures offers considerable challenges. This fact is particularly evident by comparison of 706 characterized membrane protein structures as of October 31, 2010 (http://blanco.biomol.uci.edu/Membrane_Proteins_xtal.html) to 68,998 protein structures deposited in the Protein Data Bank as of the same date (http:www.rcsb.org). The chasm separating these protein classes is not due to a lack of interesting membrane proteins to characterize, but due to intrinsic physical, chemical and biological properties of membrane proteins that impede structural characterization efforts. Membrane proteins, with the exception of rhodopsin, occur at miniscule concentrations in their natural sources. The high concentration of rhodopsin in the rod outer segments of the bovine eye contributed substantially to its successful crystallization and characterization [11]. Protein yield is most often improved through the use of heterologous expression systems. GPCR heterologous expression efforts have examined *E. coli*, yeast, insect cells infected with baculovirus, mammalian cells and cell-free systems with limited success as recently reviewed [31]. Nevertheless, the recently reported crystallographic characterization of a rhodopsin mutant isolated after heterologous expression in COS cells [32] and the β2-adrenergic, β1-adrenergic, and adenosine A2a receptors after heterologous expression in insect cells [12,14-16,33] indicate that efforts to isolate GPCRs from heterologous expression systems can fuel structural characterization studies. Expression problems that can occur include failure of the recombinant GPCR to connect with the translocation machinery of the host to reach the cell surface, or when overexpression of the recombinant protein overwhelms the naturally expressed translocation machinery resulting in toxicity toward the host. GPCRs that fail to reach the cell surface are found in inclusion bodies, requiring the development of refolding protocols, such as that developed for the leukotriene receptor [34], which must be specifically optimized for each new GPCR. Even when expression problems are solved, purification and detergent selection offer additional challenges. The successful crystallization of the turkey beta-1 aderenoceptor, for example, relied on the improved stability and detergent resistance offered by the turkey over the human receptor sequence, a property which was further enhanced before successful crystallization by the incorporation of six thermostabilizing mutations [15]. A recent study designed not to solve these problems, but to classify membrane protein sequences likely to serve as tractable targets for cloning, expression, and solubility as a tool for target selection in genomics efforts showed accuracies in the identification of true positives of >70% (tractable cloning targets), >55% (tractable expression targets), and >60% (tractable targets with respect to detergent solubility) [35]. Such tools may prove useful to identify which GPCR in a family of homologous GPCRs might prove the most tractable target for experimental structural studies. Alternatively, use of engineered constructs that eliminate highly flexible loops and provide interfaces for crystal contacts like the replacement of the third intracellular loop (IL3) of first the β2-adrenergic [14] and then the adenosine A_2A_ [16] receptors with T4 lysozyme may allow characterization of GPCRs that are intractable to crystallization in their wild type form.

The characterization of GPCR structures is further complicated, relative to other membrane proteins, by additional factors. The first complication unique to GPCRs is the conformational equilibrium involving multiple protein conformations, and the sensitivity of this equilibrium to ligands and coupled proteins [13] Only rhodopsin has the advantage of a covalently bound ligand that stabilizes a single conformation, the inactive form, of the receptor. Crystallization of a single conformation of first the β2-adrenergic [12] and subsequently the β1-adrenergic [15] and adenosine A_2A_ [16] receptors were promoted by the use of highly potent ligands, either inverse agonists or antagonists, during purification and crystallization. It remains to be seen if ligands that stabilize other conformations of these receptors can be utilized to characterize other protein conformations. Regardless, a truly detailed understanding of the structure of a single GPCR must include dynamic information. The second complication for GPCR structural studies is the occurrence of both homodimers and heterodimers based on studies utilizing both heterologous overexpression and endogenous expression [36]. Allosteric coupling between the protomers in such dimers has been clearly demonstrated [37], indicating that dimerization has structural and functional consequences.

Given these numerous challenges inherent in the attempt to experimentally characterize the structures of GPCR family members, it is not surprising that a broad variety of indirect methods have been used to gain important structural insights. A sampling of these techniques and the resulting structural insights is described in the next section, followed by a section focused on the recent successes using direct structural studies. We end with perspectives on GPCR structures in rational drug design.

## 2. Indirect Reflections of GPCR Structure

### 2.1. Insights from Pharmacological Studies

**Figure 1:**
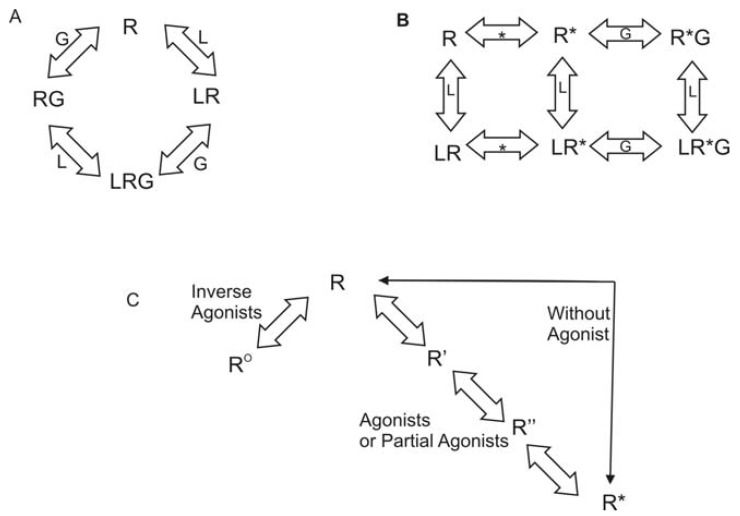
Ternary Complex Models. Panel A illustrates the classic ternary complex model [38] The receptor (R) interacts either with the ligand (L) or the G protein (G). The activated complex is generated by interaction with the remaining component resulting in LRG. Panel B illustrates the Extended Ternary Complex model as proposed by Samama *et al.*[39] The receptor can either interact with the ligand to form LR or isomerize to form R*. R* can either interact with G or with L. The two possible complexes then interact with the remaining component to form LR*G. If R interacts with L prior to isomerization, then the conformation of R will result from interaction with L generating LR*. LR* then interacts with G to form the activated complex LR*G. Panel C illustrates the method of receptor activation as proposed by Gether and Kobilka [40] In this model, the receptor is in a neutral conformation that can interact with either agonists or inverse agonists. Agonists would shift the equilibrium towards the activated complex and inverse agonists would shift the equilibrium towards in inactive complex. Partial agonists would stabilize an intermediate that could then change conformation to the activated complex. This model still allows for activation of the receptor without any ligand accounting for basal activity.

Rhodopsin is unique among all GPCRs since the ligand is covalently bound to the receptor. Reversible ligand binding is the norm throughout the remainder of the GPCR superfamily. As a result, many theories have been developed regarding activation of GPCRs other than rhodopsin. As early as 1980, researchers have explored the question of agonist-specific conformational states of GPCRs. The conceptual models generated varied from simple collision to a ternary complex [38]. Lefkowitz and coworkers used a variety of agonists and partial agonists to test four distinct models. The models were: (1) two non-interconvertible sites; (2) cyclic (allosteric) model; (3) divalent receptor and (4) ternary complex. They argue that only a ternary complex fits the data including intrinsic activity of the receptor. In this model, the receptor can interact with a ligand (L) or with the G protein (G) to form LR or RG. Then either complex can interact with the remaining component to generate the active complex, LRG. This model is shown in Figure 1A. Lefkowitz *et al.* later modified this model to include an explicit isomerization of the receptor to an active state [39]. In the revised model, the receptor can interact with a ligand (L) or could isomerize to the active state (R*). If it has interacted with the ligand, it can then isomerize to the active state. If it has isomerized, it can either interact with the ligand or with the G protein (G). All complexes eventually resulted in an activated receptor LR*G complex. This model is shown in Figure 1B. Gether and Kobilka further modified the ternary complex model to redefine R. Their starting ensemble of conformations has no preference for agonists or antagonists. R can transition to at least two other states, R^o^, which has a higher affinity for antagonists, and R*, which has a higher affinity for agonists [40] They also proposed the possibility of a series of states between R and R* that would account for partial agonists and sequential binding. To account for basal activity, this model does allow for activation of the receptor without an agonist being present. This model is summarized in Figure 1C.

### 2.2. Insights from Spectroscopic Studies

An early conceptual model of GPCR activation indicated that rigid-body movement of the 6^th^ transmembrane domain (TM6) occurred. This hypothesis was founded on studies using cysteine mutants of bovine rhodopsin [41,42] Eight single mutants were expressed and reacted with cysteine specific reagents to introduce either nitroxide spin labels for electron paramagnetic resonance studies to investigate distance changes between labeled sites upon activation or fluorescent groups to investigate environmental changes at single sites upon activation. Both methods suggested movement of TM6 after activation. A more precise timeline for the conformational changes in rhodopsin during the activation process has been established using azide probes introduced genetically using non-natural amino acid mutagenesis [43,44] Initial small rotations or tilts in TM segments 5 and 6 as early as the inactive Meta I state are consistent with the infra-red difference spectra, and are followed by more substantial rigid-body motions of these same segments.

Fluorescent labeling has been used as a staple technique to determine key structural changes in GPCRs [45-48]. A cysteine reactive fluorescent probe was used to study the β_2_ aderenoceptor [46]. Reversible changes in fluorescence consistent with a single binding domain and the response correlated to activity of a series of agonists. It was also noted that antagonists affected fluorescence resulting in the proposal that the resting state and the inactive state were not identical. Similar experiments were later performed by the same group with slightly different interpretation [47,48] In their more recent experiments, they covalently attached the fluorophore and determined that in the absence of any ligand, the β_2_ aderenoceptor exists in one conformation but that flexibility exists in that structure, giving a dynamic ensemble of closely related structures. They concluded that an antagonist does reduce flexibility, but does not change the conformation as they had previously reported. Both experiments led to the conclusion that in the presence of an agonist, the response correlates to activity.

Baneres *et al*. combined fluorescence emission with circular dichroism (CD) spectroscopy to examine the serotonin 5-HT_4(a)_ receptor [45] These experiments provided the first direct evidence for involvement of the second extracellular loop of 5-HT_4(a)_ in the binding and/or conformational changes induced upon binding of full, partial and inverse agonists using the C93-C184 disulfide bridge as a near-UV chromophore for CD studies. They investigated the conformational changes that resulted from treatment with an agonist, a partial agonist, an inverse agonist and a neutral antagonist on this mutant receptor using difference spectroscopy, in which the spectra for the isolated ligand and receptor were subtracted from that of a ligand-loaded receptor. An agonist, partial agonist and inverse agonist all showed clear differences between bound and unbound receptors, in contrast to the antagonist, which did not. This data was further confirmed using fluorescence of different Trp residues. The partial agonist had a decrease in intensity of fluorescence and the full agonist had an increase in intensity and the inverse agonist was similar to the unoccupied mutant receptor. A more recent study used reductive amination to introduce ^13^C-methyl groups as nuclear magnetic resonance (NMR)-active nuclei at lysine residues [49] NMR studies of receptors with no ligand, an inverse agonist, an agonist, and an antagonist indicate that spectra in the presence of antagonist match that in the absence of ligand, and that agonist binding induces either added mobility of K305 in extracellular loop (EL) 3, or breakage of an ionic interaction between K305 and D192 in EL2. Spectral differences in the presence of inverse agonist were consistent with a stabilizing interaction between K305 and F193 that was unique to the inverse agonist-bound receptor. These data provided further support for the existence of a spectrum of GPCR conformations, each giving differing levels of activity.

NMR has also been applied to investigate the properties of ligands when bound to GPCR. Such studies have been recently reviewed [50] and have demonstrated ligand-receptor interaction sites, differences between bound and free ligand conformation, as well as changes in ligand ionization state upon GPCR binding. More recently saturation-transfer difference NMR has been applied to demonstrate specific ligand binding to a GPCR, and subsequent transfer-NOE (INPHARMA) experiments were used to investigate how two ligands overlap within the same GPCR binding pocket [51]. The atoms from the two ligands sharing common receptor interactions allowed discrimination between candidate pharmacophore models for agonist activity at GPR40, and show promise for substantial impact on GPCR-based drug design and discovery.

### 2.3. Insights from Mutagenesis Studies

Site-directed mutagenesis has been extensively utilized in GPCR systems to investigate a variety of topics including constitutively active mutants (CAM), specific domains/motifs, ligand binding, receptor conformations upon binding agonists, antagonists, inverse agonists and the G-protein partner. The specific mutagenesis methods reviewed here are limited to Scanning Alanine Mutagenesis (SAM) and Scanning Cysteine Accessibility Method (SCAM).

Scanning Alanine Mutagenesis (SAM) was developed by Cunningham and Wells to determine the key side chain interactions between a receptor and ligand in the human growth hormone system [52]. In alanine-scanning mutagenesis, a series of mutants are generated that modify individual residues sequentially to alanine. The mutant receptors are tested for binding affinity and activity. If the activity or binding is changed, the mutated amino acid is interpreted to play some role in binding or activation of the receptor. Expression levels are closely monitored to ensure mutant receptors are present at near normal levels and have been transported to the cell surface. Alanine replacement is predicted to cause the least disruption in the main-chain conformation (unlike glycine and proline) since the amino acid is truncated at the beta carbon. It is also expected that replacement by alanine will not impose drastic electrostatic or steric constraints on the position mutated. This method is time consuming, but generates information regarding a protein when very little information is known about the active site or overall structure. However, interpretation of mutagenesis data is complicated by the possibility that a gain or loss of function could be attributed to either a local interaction of the mutated residue or a global structural effect [53].

SAM has been used in numerous GPCR systems and receptor locations. This method has been used to examine: activation [54-56], cell surface expression [57], binding of ligands [57-61] G-protein interactions [62,63] and various structural features [59,62-71]. The systems studied are just as widely ranging including: CXCR4 [61], parathyroid hormone receptors [67], corticotrophin-releasing factor receptor [68], aderenoceptors [64], histamine receptor [54], muscarinic cholinergic receptor [62,71] serotonin receptor [65], adenosine receptors [59,69], and the lutropin receptor [72].

SAM has been productively applied to define the roles of various domains and motifs of GPCRs in activation. By 1994, Moro had published two papers regarding the importance and function of the DRY motif in the muscarinic cholinergic receptors [62,71]. This group determined the effect of single point mutations in the intracellular loops that impeded G-protein binding and receptor internalization and sequestration. Parrish *et al*. also investigated the E/DRY motif, in the context of the *Saccharomyces cerevisiae* G-Protein-Coupled α-Factor Receptor, which lacks this motif. They found that a similar activation mechanism occurs, extending the role of the intracellular end of TM3 to GPCRs outside of class A [63]. They employed SAM and molecular modeling to examine the TM3 and the second intracellular loop. Mutation of one residue that aligns with the D of the DRY motif resulted in a CAM. Molecular modeling supports the prediction that the residues corresponding to the DRY motif would be in close enough proximity to each other to cause helical movement if one was mutated. SAM has also been used to study the role of the extracellular loops in ligand binding and receptor structure. Gkountelais *et al.* investigated the interactions of the highly flexible loops of the corticotrophin releasing factor receptor with peptide ligands [68]. The role of cysteines in disulfide bridges on the extracellular surface of GPCRs was also defined prior to the publication of the crystal structure of bovine rhodopsin, proving the value of the technique [69,73]. SAM has also been employed at the intracellular loops and carboxy terminus in class A [74], class B [67], and class C [66] GPCRs. Numerous studies have utilized SAM to identify residues involved in agonist recognition. Ward *et al.* examined seven alanine mutants in TM 6 and evaluated surface expression, binding of agonists and antagonists, and signaling efficacy [75]. They were able to determine residues that are likely to interact with agonists and antagonists and residues that are needed for receptor activation. Interestingly, these residues correspond to residues predicted for similar functions in other GPCRs based on traditional mutagenesis results [76-78]. Recent use of SAM has been to increase thermal and detergent stability [59,64]. In these studies, the protein was modified to increase stability of either the agonist or antagonist bound structures of adenosine and beta aderenoceptors. This approach will likely lead to major advances in structure elucidation of GPCRs.

**Scheme 1:**
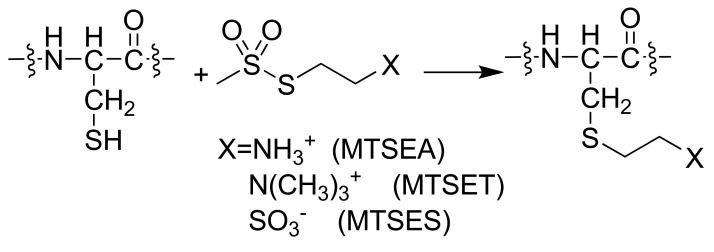
SCAM reaction. The sulfur of the amino acid sidechain is able to react with the sulfhydryl-specific reagent creating a disulfide bond when exposed to aqueous solvent. If this reaction takes place in the binding domain, the binding affinity of the ligand should decrease. Changes in the amount of reagent that reacts between inactive and active states can indicate the movement of a helical domain.

SCAM [79] analysis was first applied in the GPCR superfamily to the dopamine D2 receptor [80]. This method utilizes a combination of site-directed mutagenesis and subsequent reaction with sulfhydryl-specific reagents. Polar derivatives of methanethiosulfonate (MTS) are used, such as positively charged MTS ethylammonium (MTSEA), MTS ethyltrimethylammonium (MTSET) and negatively charged MTS ethylsulfonate (MTSES). SCAM requires wild type response after treatment of either the parent receptor or a cysteine-free mutant with sulfhydryl-specific reagents. Mutant receptors with single additional cysteine residues are expressed and ligand binding is determined with and without MTS derivative treatment. Normal binding in the absence of an MTS derivative indicates the receptor structure is unaffected by the mutation. If the residue that was replaced faces a solvent accessible area then the sulfhydryl-specific reagent will react with the S-H of the cysteine (Scheme 1). If the reagent has reacted with a cysteine in the binding crevice, it should decrease binding. If binding is unaffected, then interpretation must be done with caution. The cysteine is either not accessible to the solvent, or the mutation site is distant from the binding site. The technique has been used in numerous GPCR systems as reviewed by Javitch [81]. The method provides interesting insights into conformational differences between active and inactive GPCR conformations when applied to pairs of wild type (WT) receptors and their CAM. The difference between cysteine accessibility in WT and CAM backgrounds reflects the conformational change that occurs upon receptor activation. One interesting observation from SCAM studies in WT, partially, and completely activated CAM backgrounds in both the α1B-adrenergic [82] and μ-opioid [83] receptors is that sensitivity of ligand binding to MTS derivative treatment for cysteine mutations at certain sites (3.36 [82] and 7.38 [83]) paralleled the degree of constitutive activation. Additionally, the degree of conformational change upon activation as a function of TM helical segment can be inferred from a series of publications from the Leduc and Guillemette groups that systematically compared SCAM in WT and CAM backgrounds for the angiotensin II type 1 receptor [84-89]. These studies in aggregate show few differences in cysteine accessibility in WT and CAM backgrounds when cysteine mutants appeared in TM1 [85], TM4 [85], and TM5 [86] in contrast to very substantial differences when cysteine mutants were placed in TM2 [87], TM3 [89], TM6 [88], and TM7 [84]. On a smaller scale, TM6 was also highlighted as showing conformational differences between WT and three different CAM of the β2-aderenoceptor, as ligand binding to the WT receptor was insensitive to MTS derivative treatment, but all three CAM required mutation of the endogenous cysteine at position 285 (6.47) to serine in order to show similar insensitivity [90-92].

While crystal structures of GPCRs are becoming more prevalent, the use of mutagenesis methods such as SAM and SCAM will continue since these techniques can be applied at lower cost and faster pace to a larger cross-section of GPCR family members, and are additionally able to examine dynamic processes. These techniques allow examination of activation, helical movements upon binding a variety of ligands, specific amino acid interactions and how those micro environments change over time.

## 3. Direct Reflections of GPCR Structure

### 3.1. Crystallographic Structures

The first crystallographic structure of a class A GPCR, rhodopsin, was reported in 2000 (PDB ID 1F88) [93]. Seven additional years were required before the first crystal structures were reported by two different research groups of a GPCR activated by a noncovalently-bound ligand, the β2-aderenoceptor (PDB ID 2R4R, 2R4S, 2RH1) [12,14,33,94]. Solution of these structures relied on two techniques to produce a homogeneous population of receptors with sufficient interactions to produce diffraction-quality crystals. First, both have an inverse agonist, carazolol, bound to stabilize the protein in a single conformation. Second, intracellular loop three was either replaced with T4 lysozyme [14] or bound to a monoclonal antibody [95] to reduce its flexibility. The former strategy has also been applied characterize the crystallographic structure of another GPCR, the adenosine A2a receptor [16]. The transferability of this strategy suggests that additional GPCR structures will be reported with smaller time lags than that between the report of the first rhodopsin structure and the β2-aderenoceptor structures. A third strategy has recently emerged, in which thermostabilization of the turkey β1-aderenoceptor [64] allowed its successful characterization by x-ray crystallography [15]. Although only four different full-length examples of GPCR family members have been published to date, a number of interesting comparisons can be made from the reported structures. First, rhodopsin has been characterized in multiple photostates (Table 1) as well as in the presence and absence of 11-cis-retinal. These structures allow comparisons of different crystallographic forms of a single protein. Second, four different GPCR family members have been characterized, allowing analysis of the similarities and differences among them.

#### 3.1.1. Conformations of a single protein

Rhodopsin has long been an object of research due to its availability, high concentration in natural sources, and central role in the biologically important process of visual signal transduction. The first atomic-resolution crystallographic structure of rhodopsin was reported in 2000, leading to numerous additional structures as shown in Table 1. These structures include several examples of the ground state, dark-adapted form with the covalently bound inverse agonist, 11-cis retinal. Also included in this list are several photoactivated forms in which the original 11-cis retinal has photoisomerized to the agonist, all-trans retinal.

**Figure 2:**
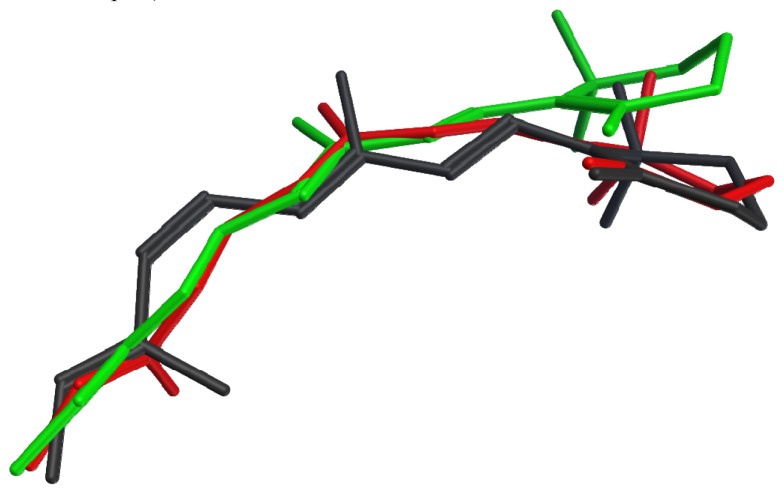
Retinal chromophore conformations in superposed rhodopsin crystal structures 1F88 [93] (dark grey, dark-adapted), 2G87 [101] (red, bathorhodopsin), and 2HPY [102] (green, lumirhodopsin).

None of the crystallized photoactivated forms provides a high-resolution structure for metarhodopsin II, the final deprotonated active state. However, structures of two earlier photointermediates, bathorhodopsin and lumirhodopsin, provide concrete confirmation of the timeline of structural changes in the retinal chromophore, many details of which had been initially proposed based on spectroscopic studies. Figure 2 shows that the structural differences between the retinal chromophore in the ground state (dark grey) and in bathorhodopsin (red) include isomerization of the bond between carbons 11 and 12 from cis to trans, and distribution of the effect of this isomerization into small changes throughout the acyclic portion of the chromophore, with almost no translational movement of the β-ionone ring. The strain due to curvature in the unsaturated linear chain is relieved during the transition from bathorhodopsin (red) to lumirhodopsin (green), resulting in a maximal atomic displacement of about 2.7 Å at the end of the β-ionone ring relative to the optimally-superposed surrounding protein backbones. It is interesting to note that these changes in the retinal chromophore are accompanied by protein backbone structural differences of only 0.41 Å root mean square deviation (RMSD). The chromophore structural changes therefore precede the protein conformational changes during the activation process.

**Figure 3:**
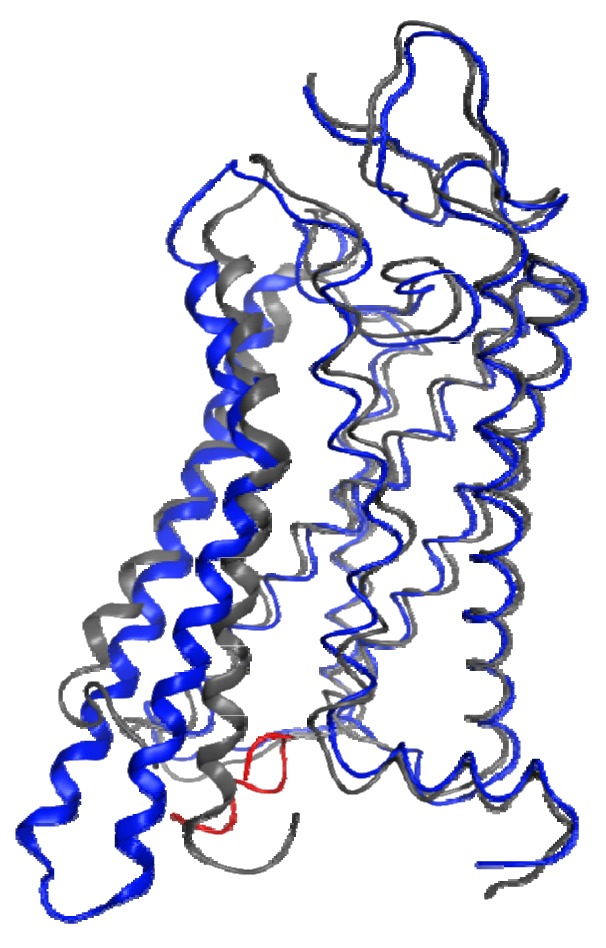
Comparison of dark-adapted rhodopsin (PDB [96] entry 1F88 [93], grey ribbon) and the ligand-free opsin complex with a Gα C-terminal peptide (PDB [96] entry 2DQB [107], opsin: blue ribbon, Gα C-terminal peptide: red). Superposition of helical segments 1-4 and 7 were optimized and these segments and helix 8 are shown as thin ribbons. Helices 5 and 6 are shown using wide ribbons.

In the absence of a metarhodopsin II crystal structure, the recent structure of a Gα C-terminal peptide complex with opsin, the ligand-free apo-protein form of rhodopsin, provides the best insights into the structural changes that occur within the protein during the activation process. Figure 3 shows a superposition of helical segments 1-4 and 7 of this opsin structure onto a dark-adapted rhodopsin structure with a 1.56 Å RMSD. This figure highlights the profound backbone structural differences that are largely isolated to TM6 (4.51 Å RMSD in this superposition) and TM5 (2.41 Å RMSD in this superposition). These structural changes are observed due to the lack of the ground-state ionic lock between R3.50 and E6.30, which tethers the sixth helix to the third in the dark-adapted state. Different interactions occur between helices 3 and 5 (R3.50 to Y5.58) and helices 5 and 6 (E6.30 to Y5.66) as well as with the bound G-protein C-terminus. These structures confirm many previous spectroscopic and mutagenic studies pointing to the role of the ionic lock between R3.50 and E6.30 as well as substantial relative motion between the ends of helices 3 and 6 during activation.

#### 3.1.2. Comparison of different proteins

Numerous sequence comparisons among GPCRs within class A have been utilized in combination with a broad range of experimental techniques to identify the structural and functional roles played by amino acids located at various positions. The availability of crystallographic structures of four different class A GPCR now provides an unparalleled opportunity to investigate some of the underlying assumptions of such comparisons.

Comparative modeling begins with the assumption that identical amino acids in structures sharing substantial sequence identity and function will have similar structures in the corresponding proteins. The four crystallized GPCRs provide an opportunity to critically analyze this assumption. Rhodopsin (PDB ID 1F88) [93], the β2-adrenoceptor (PDB ID 2RH1) [110], the β1-adrenoceptor (PDB ID 2VT4) [15] and the adenosine A2a receptor (PDB ID 3EML) [16] have 30 completely conserved amino acids (8.9-12.2% identity). Of these 30, 28 have a mainchain root mean square deviation (RMDS) of 2 Å or less (27 that occur in transmembrane segments are shown in Table 2). It is compelling to note that all seven residues selected as helical index positions [111] due to their high conservation throughout the class A GPCR family appear in this set. This provides excellent confirmation that homology models prepared from carefully optimized alignments focused on matching the helical index positions should provide good starting points to learn about the structures of as yet uncrystallized representatives of the class A GPCR family.

**Figure 4:**
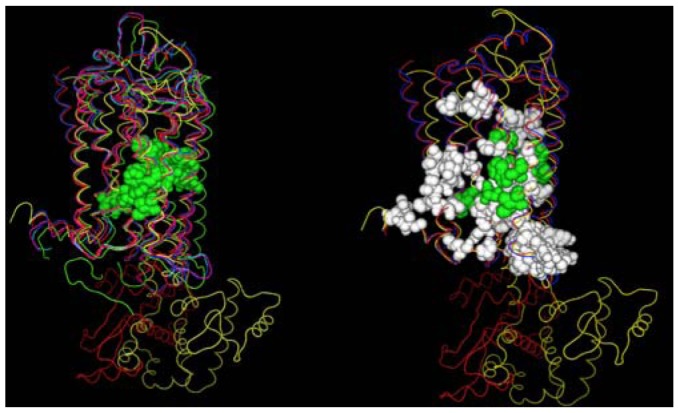
Locations of conserved contacts among class A GPCR crystal structures. *Left Panel:* Conserved contacts among dark-adapted rhodopsin (1f88 [93], green trace), ligand-free opsin (3cap [106], magenta trace), opsin with G_α_ peptide (3dqb [107], orange trace), β2-adrenoceptor(2rh1 [110], red trace), β1-adrenoceptor(2vt4 [15], blue trace) and adenosine A2a receptor (3eml [16], yellow trace) crystal structures. *Right Panel:* conserved contacts among the β2-adrenoceptor(2rh1 [110], red trace), β1-adrenoceptor(2vt4 [15], blue trace) and adenosine A2a receptor (3eml [16], yellow trace) crystal structures.

A second structural feature that can be investigated with these GPCR crystal structures, is the degree to which similar amino acids can play the same structural roles in different sequences. In this analysis we compare the interactions in which at least one partner is found in the set of amino acids conserved both at the primary and tertiary structure levels. Figure 4 shows these interactions both with (left) and without (right) several rhodopsin/opsin crystal structures. These structures demonstrate that even the most diverse sampling of currently available GPCR crystal structures share a common core of interactions involving conserved residues. Elimination of the rhodopsin/opsin structures substantially expands this set, reflecting the unique features of rhodopsin such as its covalent interaction with retinal and substantially lower sequence identity to the remaining crystallized GPCR structures. Five of six interactions highlighted in the left panel involve only one absolutely conserved amino acid sidechain, providing direct structural evidence in favor of the common expectation that conservative substitutions often produce negligible structural changes. Table 3 shows the interacting sites displayed in the left panel of Figure 4.

These comparisons indicate that the most reliable features of any comparative model of class A GPCR developed using these templates will be the intracellular ends of the transmembrane segments, a finding also reported by Mobarec, *et al.* [112] Unfortunately, this suggests that use of such comparative models to understand the binding of both natural signaling molecules and candidate therapeutics will be challenged by the greater differences in the vicinity of the binding pockets at the extracellular ends of the transmembrane segments. Thus additional methods that focus on identifying structural differences between the extracellular regions of different GPCR sequences have substantial value.

### 3.2. Fragment Methods

The combination of experimental and computational structural methods perhaps provides the best balance between accuracy and speed for the construction of sufficiently accurate models of GPCR structures for many applications. NMR spectroscopy, as recently reviewed, has been used extensively to characterize structural characteristics of GPCR segments as well as ligand interactions and dynamic properties [50]. The earliest study on conformational properties of GPCR segments focused on CD studies of TM, loop and terminal segments of the *Saccharomyces cerevisiae* Ste2p receptor [113]. Comparison of water, trifluoroethanol (TFE), dimyristoyl phosphatidylcholine (DPC) liposomes and sodium dodecyl sulfate (SDS) micelles demonstrated that synthetic peptide segments from different environments within the receptor required different solution environments to exhibit ordered structures. NMR was then applied to study the structure of individual domains of rhodopsin [114-120]. These studies provided important early insights into the structures of the aqueous-exposed loops of rhodopsin prior to the availability of an experimental structure. In many cases, the NMR data proved insufficient to define a single conformation. This is in part due to the likely flexibility of the loops within the context of the full-length receptor, as well as due to the lack of a well-folded transmembrane domain to provide a conformational restraint. NMR-based structural characterization of peptide segments from the β-adrenoceptor [121], parathyroid hormone receptor [122], angiotensin II AT_1A_ receptor [123], neurokinin-1 receptor [124,125], thromboxane A_2_ receptor [126-128], V_1A_ vasopressin receptor [129], corticotrophin releasing factor receptor 2β [130], Ste2p [131,132], the muscarinic acetylcholine M2 receptor [133], the fourth sphingosine 1-phosphate receptor, S1P_4_ [134], and the CCR5 receptor [135] then followed. Unique features of these subsequent studies include the use of conformational restraints such as an octamethylene linker between the peptide termini [122], disulfide linkages between the termini [126,127,132], or between helical loop extensions [134], use of bacterial expression systems to produce isotopically-labelled peptides to simplify chemical shift assignments [130,133,134], inclusion of coiled-coil motifs at the termini to promote self-association of the loop termini [134] and use of saturation transfer NMR to investigate interactions with binding partners [135]. Any critical scientist would naturally question the relevance of these segment conformations to the conformation of the same sequence in the context of a full-length receptor. Multiple types of validation studies have been used to address this question. One testament to relevance is the ability of intracellular segments from several GPCRs to inhibit the effect of receptor activation on downstream signaling events such as phosphodiesterase (PDE) or adenylyl cyclase activity [114,115,117,121]. The third cytoplasmic loop of the parathyroid hormone receptor has been shown to activate G proteins [122]. Fluorescence intensity changes on antagonist treatment [126,127] as well as chemical shift perturbation in response to specific agonists or agonist headgroups [125,130,134] have been used to show that extracellular segments are capable of specific ligand recognition. Table 4 summarizes segment characterization studies and the experiments utilized to validate the structures obtained as representative of the corresponding segment in the full-length GPCR.

**Figure 5:**
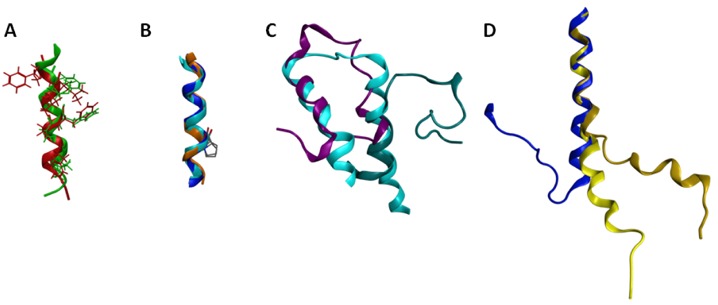
Comparisons between structures solved as GPCR fragments. Colors used are coded by GPCR and method; red (β2 fragment, 1DEP [121]), green (CB1 fragment 2B0Y [141]), orange (alpha factor receptor fragment from *S. cerevisiae*, 1PJD [144]), blue (β2-aderenoceptor crystal structure, 2RH1 [110]), cyan (rhodopsin crystallographic structure, 1F88 [93]), blue-green (rhodopsin fragment, 1EDS [139]), purple (S1P_4_ engineered fragment, 2DCO [149]), yellow (α2A-adrenoceptor fragment, 1HLL [142]), gold (DRY to IRY mutant α2A-adrenoceptor fragment, 1HOD [142]). A. Comparison of cytoplasmic loop (helix 8) structures. Hydrophobic amino acids are shown and colored to match the backbone. B. Comparison of TM6 structures. The conserved TM6 proline residue is shown as a stick model in all three structures. C. Comparison of EL1 structures. Superposition of segment structures onto 1F88 was based on best-matched structural segments involving the amino-terminal helix of 2DCO and the carboxy-terminal helix of 1EDS. D. Comparison of IL2 structures.

Several of the GPCR segments characterized using NMR have been deposited in the Protein Data Bank [94]. A list of the publicly available GPCR segment structures is provided in Table 5. Several comparisons are of particular interest either due to significant similarities or significant differences. The structures obtained for the cytoplasmic loop between the end of TM7 and the myristoylation site of the β-adrenoceptor and the CB1 cannabinoid receptor are both helical, and exhibit a root mean square deviation (RMSD) between backbone atoms of 1.6 angstroms (Figure 5A). These structures additionally place hydrophobic amino acids in common locations in the amphipathic helix, and compare well to the eighth helix observed in the rhodopsin crystal structures [11]. This similarity between cytoplasmic helical segments from two different GPCR and the full length rhodopsin structure suggests that any GPCR with an appropriately amphipathic sequence following the end of TM7 is likely to exhibit a similar structure. Another segment that shows excellent structural consistency when characterized in isolation versus within a full-length receptor is TM6 (Figure 5B). The sixth TM segment of the alpha factor receptor from *S. cerevisiae* [144] characterized in oriented lipid bilayers by solid-state NMR superposes upon the corresponding segments of the rhodopsin crystal structure [11] and the β2-aderenoceptor crystal structure [12] with a 1.2 Å RMSD. Notably all three structures show that the conserved proline residue from TM6 does not disrupt the helical structure, as also reported based on solution-phase characterization of an isolated TM6 sequence from rhodopsin in DMSO [120]. In contrast, comparison of the two characterized first extracellular loop (EL1) structures highlights substantial structural differences (Figure 5C). Figure 5C compares the rhodopsin [139] and S1P_4_ [134] EL1 NMR segment structures to the corresponding segment from the rhodopsin crystal structure [11]. The superposition demonstrates that only the carboxy terminal end of the rhodopsin EL1 segment displayed a compact structure that compared well to the full-length receptor. In contrast, the S1P_4_ EL1 segment overlapped best on the full-length rhodopsin structure at the amino-terminal end, and showed a distance between the segment ends quite similar to full-length rhodopsin structure.

**Figure 6:**
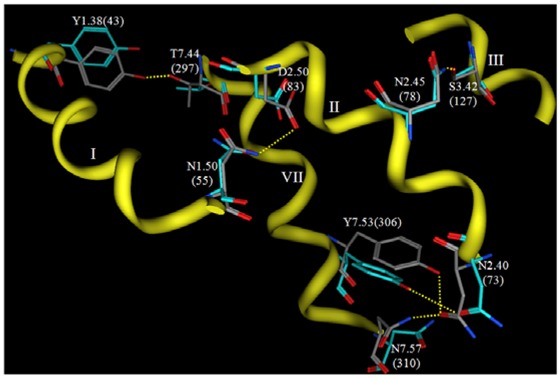
Comparison of interhelical hydrogen bonding networks in the 1997 Pogozheva 1BOJ model (grey carbons) with corresponding residues in the 2.2 Å rhodopsin crystal structure (cyan carbons). Segments of TM domains I, II, II and VII are shown as yellow ribbons.

Differences between these structures stem from a substantial number of differences both in the design of the peptide segment characterized, as well as the composition of the solutions in which they were characterized. The S1P_4_ EL1 segment utilized sequences producing an antiparallel coiled-coil on either side of the EL1 sequence as well as a disulfide bond near the center of the coiled-coil sequences to provide a structural constraint analogous to the full-length receptor. In contrast, the rhodopsin EL1 segment was completely unrestrained. The S1P_4_ EL1 segment was also characterized in a solvent system that promoted secondary structure formation, 20% trifluoroethanol. In contrast, the isolated EL1 segment of rhodopsin was characterized in DMSO due to aggregation in aqueous solution. Further studies on additional EL1 segments will be required in order to determine the true structural variability of this segment in the GPCR family. A striking difference is evident by comparison of the second intracellular loop (IL2) of the α2A aderenoceptor [142] and the β2-aderenoceptor crystal structure [12] (Figure 6D). The sequences have more than 40% identical amino acids, suggesting that their structures should share common features. The isolated segment, however, fails to form a loop, although the 10 amino acids at the amino terminal end of the α2A adrenoceptor IL2 segment superpose on the corresponding residues of the β2-aderenoceptor crystal structure with a 0.5 Å RMSD. Notably, a mutation of the conserved DRY motif in the α2A adrenoceptor segment to IRY produces a more substantial bend, improving the conformational resemblance between the isolated segment and the closely related full-length receptor. The apparent environmental dependence of the segment conformation may reflect a role in providing the flexibility needed to allow multiple conformational states of the receptor.

Figure 5 demonstrates that care must be taken when deriving insights about GPCR conformations from characterization of isolated segments. Segments with extensive short-range interactions, such as helices, seem to fold independently and can be characterized in isolation. Loop structures should be approached with carefully engineered designs that include disulfide constraints, interacting helical ends, or micelle-embedded ends, in order to derive useful structural insights. It is particularly important to provide functional validation of the segments characterized before drawing significant structural conclusions.

### 3.3. Modeling

Since GPCRs are extremely difficult to crystallize, researchers have utilized computational techniques to address difficult questions regarding ligand-protein interactions [150], structural requirements for binding [151], helical packing interactions [152], and movement of helical domains [153]. These studies have utilized either ab initio [153-157] or homology modeling of the protein.

Homology modeling studies have used a variety of template structures including bacteriorhodopsin [158,159], the theoretical model developed by Pogozheva (PDB ID: 1BOJ [152]) [160-162], the bovine rhodopsin crystal structure published in 2000 (PDB ID 1F88 [93]) [61,163-169], the rhodopsin structure published in 2004 (PDB ID 1GZM [98]) [170], and the crystal structure of the β2 aderenoceptor (PDB ID: 2R4R [33]) [171-173]. Recently groups have begun to address the issue of quaternary structure within the lipid bilayer [61] or homo or heterodimers [174,175].

Early homology modeling studies used the crystal structure of bacteriorhodopsin as a template for various GPCRs [176-180]. Bacteriorhodopsin was crystallized at 3.50 Å resolution in 1995 (PDB ID 2BRD) [181]. The resolution was later refined to 2.3 Å (PDB ID 1BRX) [182] and finally to 1.55 Å. (PDB ID 1C3W) [183] These structures were widely used to develop computational models of GPCRs due to the lack of a more closely related structure. The lack of overall homology, the differences in helical packing and arrangement as well as the difference in function soon called this practice into question [184,185].

A new era of computational modeling of GPCRs began with the publication of an experimentally-guided theoretical model for bovine rhodopsin. This model was developed using an iterative refinement process and allowed modeling of GPCRs using a GPCR template. Pogozheva, Lomize and Mosberg published a structure for the transmembrane domains of bovine rhodopsin (PDB ID 1BOJ) [93] developed with an all-trans-retinal ligand using distance geometry and hydrogen bonding calculations. Pogozheva *et al*. used the electron microscopy coordinates of the helices of frog and bovine rhodoposin to determine the spatial arrangement of the seven TMD helices. The sequences of 410 GPCR sequences were aligned from four subfamilies; peptide, protein, opsins and cationic amine receptors. The initial model was generated using limited mutagenesis and cross-linking data for distance constraints. An iterative distance geometry refinement was employed to refine this structure and to develop a series of interhelical side-chain hydrogen bonds resulting in an average model for each TMD. They assumed that hydrophilic conserved residues were likely to be inward facing and would be important for proper protein folding and interhelical hydrogen bonding as originally proposed by Zhang and Weinstein [186]. These residues defined a lipid inaccessible core and this helped to delineate the rotation and insertion depth in the membrane. Lowest energy helical-coil calculations were used to determine the seven helical domains. Each predicted helix was slightly longer than those predicted by Baldwin [187]. In addition, the rhodopsin model lacked the helix 8 predicted by Mosberg *et al*. (residues 311-320) [168], observed by Yeagle *et al*. using NMR studies of peptide fragments [188], and confirmed in the crystal structure by Palczewski [93] Even considering these differences, this model was a significant improvement over other atomic level models in that the helices were no longer rigid and side chains were used in hydrogen bonding rather than randomly assigned positions.

Now that crystallographic structures of rhodopsin have been solved at atomic resolution, it is interesting to compare such a carefully-developed and refined model to the experimental structure. Figure 6 shows a comparison of the interhelical hydrogen bonding networks in this structure versus the same sidechains in a rhodopsin crystal structure.

Both structures display hydrogen bonds from N2.45(78) to S3.42(127) and from Y7.53(306) to N2.40(73). The hydrogen bond from N1.50(55) to D2.50(83) is evident in the theoretical model, but not in the rhodopsin crystal structure due to reversed oxygen/nitrogen positions in the terminal amide. This hydrogen bond is evident, however, in recent opsin crystal structures, so this hydrogen bond prediction is quite likely to be correct. Two hydrogen bonds predicted by the theoretical model are not reflected in the crystal structure, from N7.57(310) to N2.40(73) and from Y1.38(43) to T7.44(297). Overall, the predicted hydrogen bonding networks show impressive correlation with the crystal structure given the low-resolution experimental information that provided only helical tilts and packing geometries. Many groups have used this model as a template to develop homology models of various GPCRs [152,161,163,189-195] The validity that these systems have had in predicting agonist receptor interactions suggests that this template is still suitable to develop models for the active state of class A GPCR.

The publication of GPCR crystal structures has dramatically altered homology modeling of this receptor class. Since this receptor class contains more than 800 members and sequence identity varies greatly, it is clear that one template will not suffice for modeling the entire class. To increase the difficulty of this endeavor, GPCRs likely exist in multiple conformations. The majority of crystal structures likely represent the inactive state since they are crystallized with bound antagonists or inverse agonists. Based on these challenges, investigators have attempted to determine the applicability of various template structures. The publication of the first bovine rhodopsin crystal structure (PDB ID 1F88) [93] generated great excitement in the modeling community as it constituted the first atomic-resolution experimentally-characterized template for modeling GPCR family members. This structure was used in a unique experiment by Bissantz [196] that used a homology model for virtual screening. Three models for different families (dopamine D3, muscarine M1 and vasopressin V1a) of GPCRs were developed and then used in virtual screening that contained both agonists and antagonists for the three receptors. The hits contained only antagonists and the authors concluded that the 1F88 structure was suitable for developing models useful in virtual screening with the limitation of identifying antagonists only. The recent publication of the human beta 2 aderenoceptor crystal structures has identified both similarities and differences with the rhodopsin structure. The differences suggest potential problems using rhodopsin as the basis for developing models of other GPCR. To evaluate strengths and weaknesses of rhodopsin-based homology models, Costanzi compared rhodopsin-based homology models of the human beta 2 aderenoceptor to the crystal structure of the β2 receptor (2rh1) [170]. He concludes that models approximate interactions but cannot detect all receptor-ligand interactions. He also suggests that models can be improved by incorporating published biochemical information such as known disulfide bonds in the modeling process. The results from this study were also improved by changing the conformation of Phe290 from trans to gauche+. This conformation was only detected in the crystal structure and would therefore be difficult to incorporate into new models. However, such a conformational difference might be captured by the typical current practice of docking against multiple protein structure generated (for example) by molecular dynamics simulations.

The value of GPCRs as targets for therapeutic intervention in a vast array of human diseases has stimulated a variety of specialized *ab initio* modeling algorithms focused in part, or solely, on generating models of GPCRs suitable to guide drug discovery programs. Other methods applied generally to membrane proteins have been reviewed elsewhere [197]. One of the earliest specialized GPCR modeling methods is the MembStruk algorithm, which requires an alignment of the GPCR family to guide selection of transmembrane alpha-helical segments, and an electron density map used to orient the helical segments [156,157]. This algorithm uses helical hydrophobicity moments to initially orient the helical segments in the bundle, but performs an iterative coarse-grained rotational search followed by rigid body molecular dynamics of the lipid-solvated helical bundle to optimize the helical packing. A later report details the PREDICT algorithm, which requires only an estimate of the transmembrane helical segments, without requiring well-defined ends [198]. This method uniquely performs the rotational orientation of the helical segments in two dimensions, and includes not only a hydrophobicity moment, but also consideration of the common occurance of aromatic residues at the helical interface to form interhelical π-stacking interactions. GPCR models constructed using the PREDICT algorithm were utilized as *in silico* screening tools and successfully identified novel hits with binding affinities less than 60 nM for 5-HT1a, NK1, 5-HT4, and dopamine D2 receptors [199]. The only target tested for which a nanomolar hit was not identified was the CCR3 receptor, which produced a novel best hit with a 12 μM K_i_ [199]. Two other methods were reported in 2006, extension of the Rosetta methodology to helical membrane proteins [200], and TASSER [201]. The extension of Rosetta to membrane proteins is the first true *de novo* protein folding algorithm applied to GPCRs, although TM region prediction is utilized as input to define the approximate membrane normal vector. This extension required significant alteration to the potential energy function, including definition of environmentally-specific residue interactions with both the environment and other residues. The Rosetta method was tested on 12 crystallographically-characterized partial and full-length membrane proteins, including rhodopsin [200]. While the method was in some cases able to predict 100% of the residues within 4 Ǻ of the native structure, this was not the case for rhodopsin, in which only 33% of the residues were predicted within 4 Ǻ of the native structure. Its applicability to develop GPCR models for *in silico* screening applications has not been tested. The TASSER method, in contrast, combines threading for fragment template identification, with a fragment assembly algorithm. TASSER is best characterized therefore as a hybrid threading/homology modeling algorithm. TASSER was applied to model the 907 putative human GPCRs [201]. The resulting models are freely available for noncommercial purposes (http://www.bioinformatics.buffalo.edu/GPCR). TASSER was validated using 38 crystallographically-characterized membrane proteins, with exclusion of templates sharing more than 30% sequence identity with the target. TASSER showed inconsistent results, with root mean square deviation (RMSD) versus native structures ranging from 1.86 Ǻ to 40.35 Ǻ. The three best-ranked models of the β2-aderenoceptor compare reasonably well to the recently reported crystal structures, with all-atom RMSD values of 4.6 Å, although the helix in the second extracellular loop was not accurately predicted. This variability is likely due to the relatively inconsistent number of proteins in a given fold family that have been characterized, and suggests that TASSER models should be used only when they can be validated by comparison to available experimental data. However, of the *ab initio* model construction methods, TASSER and Rosetta are the only methods that can be applied through web interfaces (see http://cssb.biology.gatech.edu/skolnick/webservice/tasserlite/index.html and http://robetta.bakerlab.org/, respectively).

Other algorithms focus on subsets of the GPCR structure prediction problem. Algorithms designed to identify the residues located in transmembrane segments provide excellent results as thoroughly reviewed by Punta *et al.* [197] As noted previously, several of the methods utilized to construct GPCR models rely on prior identification of the residues comprising each transmembrane segment. A second subset of the GPCR structure prediction problem is prediction of whether residues in a transmembrane segment are exposed to lipid, or buried against other transmembrane segments. Park *et al.* recently reported application of a support vector classifier to the burial prediction problem [202]. The classifier provided 78% accuracy on a benchmark data set composed of 3,138 residues from transmembrane regions of 43 protein chains. Finally, a method has recently been reported that uses knowledge-based pair potentials to predict helix pair geometries [203]. Distance-dependent pair potentials were derived using a training set of 71 crystallographic membrane protein structures and applied to the problem of rigidly docking transmembrane helix pairs from 58 test proteins [203]. A critical finding from this study is that the native arrangement was found in the largest cluster, rather than at the lowest energy, suggesting the means by which the optimal configuration can be identified in cases where the native structure is unknown.

Two characteristic problems associated with characterizing GPCR conformations are not specifically addressed by the previous methods. The first neglected problem is that of loop modeling. The second is the issue of oligomerization. Both of these issues have been recently reviewed [197,204]. Several studies on loop modeling have appeared after these reviews. Gao and Stern compared the ability of several energy functions to select the native loop conformations from a set of decoy conformations generated by molecular dynamics simulations [205]. They concluded that current energy functions, while not perfect, are applicable to the modeling loops of membrane proteins. Thus the key problem that must be solved is how to efficiently sample the conformations of each loop, with consideration of both the constraints produced by the transmembrane region, as well as the constraints produced by the remaining two loops and the terminal segment on the same face of the membrane. The loop modeling methods reported to date all sample conformations of only a single loop, and therefore must be applied in iterative cycles if loop-loop interaction effects are to be considered.

The current decade has been marked by the appearance of modeling studies that more accurately reflect the surrounding environment of GPCRs, in particular, the surrounding lipid membrane. Trent *et al*. published a model of the CXCR4 receptor in a lipid bilayer in 2003 [61]. They developed their model using the 2,000 crystal structure of bovine rhodopsin as the template. The model was inserted into a lipid bilayer with water molecules present. A sequence of constrained dynamics lead to the totally unrestrained molecular dynamics of all atoms for 4 ns. An inverse agonist (T140) and a weak partial agonist (AMD3100) were each separately docked in the resulting receptor-water-lipid complex. This system rationalized the mutagenesis data for both the partial agonist and the inverse agonist. One question plaguing the field of GPCR modeling is oligomerization. In 2006, Weinstein *et al*. published two studies on the effects of rhodopsin dimerization in a lipid bilayer. In the first study, a monomer and a dimer were inserted into the lipid bilayer to determine stability. The α carbon backbone had fewer fluctuations in the dimer versus the monomer. In the second study, they presented a unique strategy for development of an active conformation of rhodopsin using distance constraints. A series of six active models were developed with constraints from various experimental studies using experimental data from a variety of GPCR systems. Dimer models were created by placing helices 4 and 5 in contact symmetrically. Each dimer was inserted into the lipid bilayer for a one nanosecond molecular dynamics simulation. This study observed non-symmetric movement of several helixes resulting from interaction of the dimer interface. Specifically they predict that 2.93/5.61 and 6.26/7.59 should have increased cross-linking These predictions would be interesting candidates for experimental testing of the dimer importance in modeling. Much longer simulations than previously reported (microseconds) were utilized to explore the ligand entry pathway of the endogenous lipid agonist of the second cannabinoid receptor (CB2), 2-arachidonylglycerol [206]. The agonist was observed to initiate entry into the GPCR by lateral diffusion from the outer leaflet of the surrounding lipid bilayer, with concomitant conformational changes in the intracellular loops consistent with the changes expected upon receptor activation.

## 4. Perspectives on GPCR Structures in Drug Design

Both indirect and direct GPCR structure characterization studies have had tremendous impact on the optimization and discovery of GPCR-targeted ligands. Further characterization of GPCR structure and dynamics will continue to be of broad interest due to the involvement of these receptors in such a vast array of physiological and pathophysiological pathways. The currently available GPCR crystal structures demonstrate that the largest structural differences are localized to the extracellular ends of the transmembrane segments and the loops connecting these transmembrane segments. The most valuable direct structural characterization studies will therefore focus on elucidating the structures of a diverse subset from the GPCR superfamily defined based on both length and sequence divergence in the extracellular loops. A second key value area will be the elucidation of structures for agonist-bound active GPCR conformations. Such structures will provide critical insight and guidance for the rational discovery and optimization of agonists, analogous to the most successful current applications of rational GPCR ligand discovery to date which have produced antagonists.

## Figures and Tables

**Table 1:** Crystal structures of rhodopsin available in the protein databank [96].

**PDB ID**	**Year**	**Resolution (Å)**	**Ligand**	**Reported Photostate^a^**
1F88 [93]	2000	2.80	11-*cis* retinal	
1HZX [97]	2001	2.8	11-*cis* retinal	
1GZM [98]	2002	2.65	11-*cis* retinal	
1L9H [99]	2002	2.60	11-*cis* retinal	
1U19 [100]	2004	2.2	11-*cis* retinal	
2G87 [101]	2006	2.60	strained all-*trans* retinal	Bathorhodopsin
2HPY [102]	2006	2.80	all-*trans* retinal	Lumirhodopsin
2I35 [103]	2006	3.80	11-*cis* retinal	
2I36 [103]	2006	4.10	11-*cis* retinal (unresolved)	
2I37 [103]	2006	4.15	all-*trans* retinal (unresolved)	Photoactivated
2J4Y^b^ [32]	2006	3.40	11-*cis* retinal (not modeled)	
2PED [104]	2007	2.95	9-*cis*-retinal	
3C9L [105]	2008	2.65	11-*cis* retinal (reinterpretation of 1gzm)	
3C9M^b^ [105]	2008	3.40	11-*cis* retinal (reinterpretation of 2j4y)	
3CAP [106]	2008	2.90	ligand-free opsin	
3DQB [107]	2008	3.20	ligand-free opsin complexed with Gα c-terminal peptide	
2Z73^c^ [108]	2008	2.50	11-*cis* retinal	
2ZIY^c^ [20,29,109]	2008	3.70	11-*cis* retinal	

a Dark-adapted forms were characterized unless otherwise noted;

b Thermally stable mutant;

c Squid rhodopsin, unmarked entries are structures derived from bovine rhodopsin.

**Table 2:** Amino acids conserved at both the primary and tertiary (mainchain RMSD < 2 Å) structure levels among the transmembrane segments of rhodopsin (PDB ID 1f88) [93], the β2- (PDB ID 2RH1) [110], and β1-adrenoceptors(PDB ID 2VT4) [15] and the adenosine A2a receptor (PDB ID 3EML) [16].

**TM1**	**TM2**	**TM3**	**TM4**	**TM5**	**TM6**	**TM7**
N1.50	N2.40	C3.25	A4.42	P5.50	I6.39	N7.49
L1.52	L2.46	L3.43	W4.50	L5.51	F6.44	P7.50
	A2.47	A3.47	P4.60		C6.47	I7.52
	A2.49	R3.50			W6.48	Y7.53
	D2.50	Y3.51			L6.49	P6.50

**Table 3:** Common contacts exhibited by residues conserved at both the primary and tertiary structure levels.

**Conserved position**	**Interaction partner**	**Exposure**
W4.50	S/N 2.45	Surface-exposed
W4.50	I/V 4.46	Surface-exposed
L5.51	F/V 5.47	Buried
F6.44	I/L 3.40	Buried
F6.44	W6.48	Buried
I7.52	I/M 6.40	Buried

**Table 4:** NMR structural studies on GPCR segments, organized by parent receptor.

**Receptor**	**Segment**	**Validation**	**Findings**	**Solvent**
Rhodopsin [115]	IL3	Inhibits transducin activation	30% unstructured Helix-loop-helix motif	water
Rhodopsin[114]	C-terminus	Inhibits transducin activation	β-sheet, exposure of serine residues consistent with phosphorylation sites	water
Rhodopsin[116]	C-terminal loop, C-terminus		Helical c-terminal loop	phosphate buffer, pH 5.9
Rhodopsin[136,137]	C-terminus, 7-phospho-C- terminus	arrestin-bound, induces arrestin conformational changes similar to rhodopsin	Only phosphorylated segment becomes ordered upon interaction with β –arrestin, forming compact helix blocking transducin binding	phosphate buffer, pH 6.5
Rhodopsin [117]	IL1, IL2	IL2 inhibits transducin activation	Both form similar β-turns	phosphate buffer, pH 5.9
Rhodopsin [118]	Cytoplasmic face		Individual peptide segments make intermolecular contacts in solution	phosphate buffer, pH 5.9
Rhodopsin [120]	6^th^ TM		Proline has little impact on helicity	DMSO
Rhodopsin [138]	7^th^ TM		Two structural families observed with helix-break-helix architecture	DMSO
Rhodopsin [139]	EL1, EL2, EL3		Central turns in each loop	DMSO
CB_1_ [140]	IL3	Binds to G_αi1_	Helical structure when bound to G_αi1_, mutant with reduced G_αi1_ coupling adopts turn	acetate buffer, pH 6.0
CB_1_[141]	C-terminal loop	Inhibits adenylyl cyclase activity	Extended in water, 3_10_ helix in presence of SDS micelles	water SDS
β-aderenoceptor [121]	C-terminal loop	Inhibited adenylyl cyclase activity	Amphipathic α-helix, no structural effect of different solvents	TFE, micelles, vesicles
α2A-aderenoceptor [142]	IL2		Extended helix with bend induced by DRY to IRY mutation	micelles
Parathyroid hormone receptor [122]	IL3		Linear and cyclic both unstructured in water, shorter flexible segment in cyclic than linear peptide in SDS	water SDS
Parathyroid hormone receptor [143]	N-terminal fragment		Amphipathic α-helices separated by flexible region lead into TM1 through a 90 degree turn	micelles
Angiotensin II AT_1A_ [123]	IL3	N-terminal IL3 fragment activates purified G proteins	Conformational variability of overlapping region in two fragments suggests proline ‘switch’	30% TFE
Neurokinin-1 receptor [124]	EL2	Structure consistent with photoaffinity labeling	Helices at termini and center, unstructured between	water, pH 5
Neurokinin-1 receptor [125]	N-terminus EL3	Chemical shift perturbation of EL3 in response to agonist	EL3 structure has non-associating helical termini which can be induced to fit TM template	DPC micelles
V_1A_ Vasopressin	Receptor [129]IL2	Uncompetitive inhibition of agonist binding	Structure of linear and cyclic comparable to IL2 of rhodopsin crystallographic structure, cyclic peptide exhibits more stable termini	50% TFE
Thromboxane A2 [126]	EL2	antagonist-induced change in fluorescence intensity	Terminal disulfide required for function, Two β-turns	phosphate buffer, pH 6
Thromboxane A2 [127]	EL3	antagonist-induced change in fluorescence intensity	Terminal disulfide required for function β-turn	phosphate buffer, pH 5.5
Thromboxane A2 [128]	EL1		β-turn External binding site for preliminary antagonist association suggested	phosphate buffer, pH 6
CRF 2β [130]	N-terminus	Chemical shift perturbation by peptide hormones	Short consensus repeat fold identified	BisTris (HCl) buffer, pH 7.4
Ste2p [132]	IL1		N-terminal helix Even cyclic form highly flexible	DMSO
S1P_4_ [134]	EL1	Chemical shift perturbation specific to agonist headgroup	Disulfide cross-link coupled with coiled-coil provided receptor-like conformational restraint	20% TFE
			Ordered 3_10_ helix followed by more flexible sequence	
α-factor [144]	6^th^ TM		Proline shows little impact on helicity	oriented lipid bilayer (solid)
M_3_ muscarinic [145]	IL3		Type IV and Type I turn at residues responsible for basolateral sorting	phosphate buffer, pH 6.4
Cholecystokinin A [146]	EL3	Intermolecular NOE with cholecystokinin-8 consistent with mutants	Central amphipathic helix found at zwitterionic micelle surface Ligand binding contacts at end of TM6	DPC micelles
Cholecystokinin A [147]	N-terminus	Intermolecular NOE with cholecystokinin-8 consistent with mutants	N-terminal helix followed by disulfide crosslinked β-sheet	DPC micelles
Cholecystokinin B [148]	EL3	Intermolecular NOE with cholecystokinin-8 consistent with mutants	Central amphipathic helix found at zwitterionic micelle surface Ligand binding contacts at end of TM7	DPC micelles
